# Benzodiazepine use in Spain: risks and perspectives on the current situation and proposals for their rational use

**DOI:** 10.3389/fphar.2025.1547488

**Published:** 2025-05-20

**Authors:** Carlos Roncero, Lorenzo Armenteros, Carmen Bellido-Cambrón, Amparo Bonilla-Guijarro, Emilio Gómez-Cibeira

**Affiliations:** ^1^ Health Science Faculty, European University Miguel de Cervantes, Valladolid, Spain; ^2^ Pychiatric Unit, School of Medicine, Instituto de Investigación Biomédica de Salamanca (IBSAL), University of Salamanca, Salamanca, Spain; ^3^ Islas Canarias Health Center of Lugo, Lugo, Spain; ^4^ Spanish Society of General and Family Physicians (SEMG), Madrid, Spain; ^5^ Occupational Risk Prevention Department, Peripheral Unit No. 1, General University Hospital of Castellón, Castellón, Spain; ^6^ Spanish Association of Occupational Medicine, Madrid, Spain; ^7^ School of Pharmacy, Universidad San Pablo-CEU, CEU Universities, Boadilla del Monte, Spain; ^8^ Farmacia La Milla, Community Pharmacy, Madrid, Spain; ^9^ Neurology Department, Infanta Cristina Hospital, Madrid, Spain; ^10^ Sleep Unit, Ruber Juan Bravo Hospital, Madrid, Spain

**Keywords:** sleep, insomnia, benzodiazepines, abuse, responsible use, overuse, misuse

## Abstract

Benzodiazepines are among the most widely prescribed treatments for insomnia, and can have positive effects on sleep when used for a maximum of 4 weeks. However, benzodiazepines disrupt sleep architecture, and their long-term use leads to negative outcomes, including impairments in memory and attention, increased risk of falls (particularly in the elderly) and car accidents, dependence, and addiction. In addition, stopping benzodiazepines after long-term use can result in severe withdrawal symptoms. Spain is one of the countries with the most widespread benzodiazepine use. A multidisciplinary approach is crucial for the treatment of insomnia and for preventing benzodiazepine overuse/misuse. The present study provides an updated overview of the epidemiology and use of these drugs in vulnerable populations (adolescents, older adults and people with mental disorders). We also describe deprescribing strategies used in clinical practice and present two case studies to exemplify the complexities of benzodiazepine withdrawal observed in our practice. Finally, proposals are provided for the rational use of benzodiazepines in Spain, targeted to the general population, healthcare professionals, and regulatory authorities, in order to improve the clinical management of insomnia.

## 1 Introduction

An estimated 6.0%–14.8% of all adults in high-income regions, including Spain, suffer from chronic insomnia (RAND Europe, 2023; de Entrambasaguas et al., 2023), with insomnia symptoms affecting up to 43.4% of the Spanish population (de Entrambasaguas et al., 2023). In the short-term, insomnia has a negative impact on functioning and wellbeing, causing fatigue, attention and memory problems, reduced productivity at work, and mood disturbances (Brownlow et al., 2020; Whiting et al., 2023; Gingerich et al., 2018). In the long-term, insomnia is associated with poor health-related quality of life (HRQoL) and an increased risk of a wide range of diseases, including cardiovascular disorders, mental problems, dementia, arterial hypertension, type 2 diabetes, obesity, cancer, and multimorbidity (RAND Europe, 2023; Zhou et al., 2023; Sofi et al., 2014; Hertenstein et al., 2019; Almondes et al., 2016; Johnson et al., 2021; Wu et al., 2023; Shi et al., 2020; Zheng et al., 2019; Valenzuela et al., 2022). As a result, sleep disturbances pose a high burden on individuals, healthcare systems, and society at large (Rosekind et al., 2010; Hillman et al., 2018). However, despite the severe impact of insomnia, only a small percentage of people seek medical help (Torrens Darder et al., 2021).

Benzodiazepines are a class of psychoactive drugs that have anxiolytic, sedative, hypnotic, muscle relaxant and anticonvulsant effects. They are the most commonly prescribed drugs for treating insomnia in some countries, and are also used for managing other conditions such as anxiety, alcohol withdrawal, or acute muscular pain (Soyka et al., 2023; Bounds and Patel, 2024). Benzodiazepines target GABA_A_ receptors (GABA_A_Rs), inhibiting synaptic signaling throughout the central nervous system (CNS). These drugs are effective in the acute treatment of insomnia (De Crescenzo et al., 2022), and the recommended maximum duration of treatment for insomnia is 4 weeks (Brandt et al., 2024; Riemann et al., 2023). Only in some cases may longer treatment (off-label use) be considered individually (Riemann et al., 2023) after re-evaluation of the patient (Spanish Agency of Medicines and Medical Devices, 2000). Despite the indication and recommendation of the ≤4 weeks treatment period, benzodiazepines are frequently prescribed off-label for much longer periods, ranging from several months to years (Soyka et al., 2023; Urru et al., 2015; López-Pelayo et al., 2019; Cañellas Dols et al., 1998). This may be due at least in part to limited access to therapeutic alternatives offering better efficacy and safety profiles in both short- and long-term use.

The long-term use of benzodiazepines is associated with several undesired adverse effects. It can lead to alterations in sleep architecture, with an increased time of stage 2 non-rapid eye movement (NREM) sleep and a decreased time of stages 3 and 4 NREM sleep and rapid eye movement (REM) sleep (de Mendonça Maraucci Ribeiro et al., 2023). The chronic use of benzodiazepines can also impair cognitive functions across all domains (Barker et al., 2004a), with these effects persisting even after discontinuation (Barker et al., 2004b). Additionally, benzodiazepine exposure can alter attention and reaction times, increasing the risk of falls (especially in the elderly population) and traffic accidents (Smink et al., 2010; Barbone et al., 1998), making benzodiazepines the second most frequently involved substances in traffic accidents after alcohol (Alvarez-Freire et al., 2023). The risk of work accidents also increases with prolonged use and following discontinuation (Baudot, 2024). The use of benzodiazepines before a work-related injury may increases the risk of disability after the injury (Nkyekyer et al., 2018). Patients consuming benzodiazepines, especially those with long-time use, commonly report several adverse effects including daytime drowsiness, dizziness, light-headedness, disinhibition, delirium, fatigue, anterograde amnesia, and depression (Holbrook et al., 2000; Del Rio Verduzco et al., 2023). Furthermore, benzodiazepines are known to enhance the misuse of opioid medications (Cragg et al., 2019), and their combination can result in serious risks, including death (US Food and Drug Administration, 2024).

Failure to adhere to the recommended duration of benzodiazepine treatment can result in chronic use, leading to tolerance and dependence within a few weeks (Edinoff et al., 2021). Benzodiazepine misuse is a growing public health problem that can occur for different reasons. Firstly, patients may deviate from the prescribed posology, using the medication for longer periods, at higher doses, or more frequently (Soyka et al., 2023; Urru et al., 2015; López-Pelayo et al., 2019). Secondly, benzodiazepines might be used without a prescription, obtained from friends or family members with a prescription, or from illicit sources (Votaw et al., 2019).

European clinical guidelines recommend cognitive-behavioral therapy for insomnia (CBT-I) as a first-line management option for chronic insomnia. If CBT-I proves ineffective, a pharmacological intervention can be proposed, including benzodiazepines and Z drugs for up to 4 weeks (or longer in some cases; recommendation level B), orexin receptor antagonists for up to 3 months (or longer in some cases; recommendation level A), or prolonged release melatonin in patients >55 years for up to 3 months (recommendation level B) (Riemann et al., 2023). Despite these recommendations, CBT-I is rarely used as initial treatment, and benzodiazepines and Z drugs are often prescribed instead (Torrens Darder et al., 2021; Soyka et al., 2023; Ellis et al., 2023).

The present study describes the current scenario of benzodiazepine use and abuse in Spain, a country with some of the highest rates of use and misuse globally (Ma et al., 2023), despite existing recommendations aimed at preventing misuse (Spanish Agency of Medicines and Medical Devices, 2000). Also, we describe the challenges of benzodiazepine deprescription and present feasible proposals to promote the appropriate use of these drugs for the treatment of insomnia. A multidisciplinary group of healthcare professionals (a psychiatrist, a primary care physician, an occupational medicine physician, a community pharmacist and a neurologist) with experience in the management of benzodiazepines in sleep disorders across different contexts, convened on 6 March 2024 to review the available scientific evidence on benzodiazepine use, misuse and abuse in Spain; to discuss commonly found patient situations in the management of benzodiazepines (presented here as case studies); and to develop the strategic proposals for the rational use of benzodiazepines reported herein. These strategic proposals were subsequently graded for feasibility (“moderate” or “high”) and relevance (“moderate” or “high”) by the authors via an online survey in July 2024.

## 2 Benzodiazepine use in Spain

### 2.1 Epidemiology

European countries are among the largest consumers of benzodiazepines in the world (Ma et al., 2023), with Spain ranking among the highest in usage rates (Lukačišinová et al., 2024). Research on volume per class for medicines prescribed for insomnia among European countries in 2022 showed that the sales by volume for benzodiazepines were highest in Spain, accounting for 66.3% of the total (Soyka et al., 2023). The consumption of anxiolytic and hypnotic prescription drugs in Spain has been rising since 2010, peaking in 2021 with 93.3 defined daily doses per 1,000 inhabitants per day (Spanish Agency of Medicines and Medical Devices, 2024).

The pronounced peak recorded in 2020 and 2021 might be related to the COVID-19 pandemic, which witnessed an increase in insomnia, anxiety and depression symptoms in Spain (Zhang et al., 2022; Roncero et al., 2024), with prevalence rates exceeding those of other countries like China (Zhang et al., 2022). A study of 1,673 Spanish adults found that during the lockdown, sleep quality worsened, with increased daytime sleepiness, a higher number of awakenings, and a longer duration of awakenings (Ruiz-Herrera et al., 2023). The rise in sleep problems during the pandemic was accompanied by an increase in benzodiazepine consumption (Sánchez Díaz et al., 2021; Perelló et al., 2023). A study across 75 community pharmacies in the region of Catalonia reported a significant rise in benzodiazepine use during the pandemic compared to the two previous years (Perelló et al., 2023) - a trend mirrored in other countries (Zaki and Brakoulias, 2022; Kurvits et al., 2024).

Along with the impact of the pandemic, the continuous rise in benzodiazepine use in recent years can be attributed to several factors. These include a growing trend towards low tolerance for frustration (Marquina-Márquez et al., 2022). Healthcare professionals often face pressure from patients who demand immediate solutions, which together with the limited time for appointments, underestimation of the associated adverse events, and low access to non-pharmacological resources, results in a higher prescription rate of benzodiazepines (Marquina-Márquez et al., 2022). Therapeutic inertia, a low perception of consumption risks, and a lack of knowledge regarding benzodiazepine withdrawal management might also contribute to the increase in benzodiazepine prescriptions (Vicens et al., 2014; Vázquez Canales and Frutos Fernández, 2023).

In addition to the high use of prescribed benzodiazepines, non-medical use—i.e., the use of benzodiazepines without a corresponding medical prescription—is also prevalent in Spain (Novak et al., 2016). A study conducted in Barcelona revealed that one in 11 citizens used benzodiazepines off-label, with primary care physicians and psychiatrists being the most common off-label prescribers (López-Pelayo et al., 2019). Also, a high prevalence of potentially inappropriate use of benzodiazepines has been detected in community pharmacies and nursing homes across Spain (Díaz Planelles et al., 2023; Perelló et al., 2021).

The use of benzodiazepines among the healthcare population is also important. A recent survey conducted among 1,121 healthcare professionals has found that around 30% of the respondents had sleep problems, and over 25% were using benzodiazepines (Roncero et al., 2025).

Community-based interventions have been launched in some regions to promote benzodiazepine deprescription among long-term users. One of these initiatives was the “benzoletter,” where primary care physicians sent a personalized letter to patients who had been using benzodiazepines for more than 3 months, providing information about the adverse effects of such use and a recommendation to discontinue them (Baza Bueno et al., 2020). These local initiatives have achieved some success in reducing benzodiazepine use within targeted populations (Baza Bueno et al., 2020). Nevertheless, misuse and abuse of these medications continue to be prevalent in Spain. The difficulties in instituting possible therapeutic alternatives (both pharmacological and non-pharmacological) offering better efficacy and safety profiles, and with timely access to them, might have limited the success of such local initiatives.

### 2.2 Vulnerable populations

#### 2.2.1 Adolescents

Recommendations from experts suggest treating insomnia in children and adolescents with CBT-I, limiting the use of benzodiazepines, among other drugs, as much as possible (Pin et al., 2017). However, the use of benzodiazepines among the younger populations in Spain has increased over the years, with a large part of this consumption being non-medical (Carrasco-Garrido et al., 2021a). In 2023, the prevalence of non-medical hypnosedative use among students aged 14–18 years in the last 12 months was 7.4% (Spanish Ministry of Health, 2023a). Consumption is higher among women than in men (Carrasco-Garrido et al., 2021a; Spanish Ministry of Health, 2023a; Carrasco-Garrido et al., 2021b). Specifically, 26.1% of 14- to 18-year-old females admitted to having used these substances at least once in their lifetime, 12.7% of them without a prescription (Spanish Ministry of Health, 2023a).

These figures are concerning not only due to the adverse effects of benzodiazepines but also because there is a relationship between the use of benzodiazepines and the consumption of other substances, such as alcohol, tobacco and marijuana among adolescents and younger adults (Carrasco-Garrido et al., 2021a; Carrasco-Garrido et al., 2018; Palacios-Ceña et al., 2019). Additionally, a survey of 10,824 individuals aged 15–34 years in Spain revealed that lower awareness of drug use risks and more negative health perceptions are associated with the non-medical use of benzodiazepines and Z-drugs among young people (Carrasco-Garrido et al., 2021b).

#### 2.2.2 Older adults

Benzodiazepine use in Spain, including off-label use, increases with age (López-Pelayo et al., 2019; Herrera-Gómez et al., 2018; Spanish Ministry of Health, 2023b), and is more prevalent among women (Spanish Ministry of Health, 2023b; Torres-Bondia et al., 2020). Approximately one-third of all community-residing older adults use benzodiazepines, which is one of the highest prevalence rates among this population in European countries (Lukačišinová et al., 2024). This prevalence is alarming, given that research indicates that benzodiazepine use in the elderly is associated with several negative outcomes, including dependence, an increased risk of falls resulting in fractures, cognitive impairment, dementia and mortality (Markota et al., 2016; Aldaz et al., 2021). Whether the association between benzodiazepine use and dementia is causal or whether these drugs are more frequently prescribed to patients experiencing prodromal symptoms of dementia remains unclear. However, the stronger association observed between long-term benzodiazepine exposures and the risk of Alzheimer’s disease (Billioti de Gage et al., 2014), as well as the increased risk of dementia associated with the concomitant use of two or more benzodiazepines (Tseng et al., 2020), suggest a direct association, although it does not necessarily imply causality. Also, the use of benzodiazepines has been associated with an accelerated reduction of hippocampal and amygdala volume (Hofe et al., 2024). This is despite the possibility that benzodiazepine use might also be an early indicator of conditions such as sleep problems, that are associated with a higher risk of developing dementia (Bubu et al., 2016; Shi et al., 2018).

Considering the available evidence, the potential benefits of benzodiazepines for older adults may not justify the associated risks, particularly when the patients have additional risk factors for cognitive or psychomotor adverse events (Glass et al., 2005). Clinicians should be very cautious when prescribing these medications to older patients. Some medical societies, such as the American Geriatrics Society, recommend avoiding benzodiazepines and non-benzodiazepine hypnotics in this population (By the 2023 American Geriatrics Society Beers Criteria^®^ Update Expert Panel, 2023).

Given the high prevalence of insomnia among the elderly, and the complications related to benzodiazepine use, a complete clinical evaluation/screening and alternative pharmacological and non-pharmacological options should be considered (Vázquez Canales and Frutos Fernández, 2023). Before initiating any treatment, it is important to conduct an assessment of the medical and psychiatric history, and to evaluate the sleep environment and psychosocial stressors of the patient.

#### 2.2.3 People with mental disorders

Benzodiazepines are prescribed for a variety of mental disorders, including not only anxiety and depressive disorders but also schizophrenia, where they are used to manage acute anxiety and prevent relapse after stabilization. While the short-term use of benzodiazepines for specific symptoms can be appropriate, long-term use and at higher than recommended doses remains common among patients with mental health disorders in Spain (Simal-Aguado et al., 2021). The effectiveness and potential adverse effects of these medications on the symptoms of different mental disorders are subjects of ongoing debate.

Patients with mental disorders also have a higher tendency towards addictive behaviors, a problem that is often under-recognized (Szerman et al., 2022). In turn, this addiction to substances is associated with a worse progression of underlying mental disorders. Abuse of benzodiazepines was correlated with an incomplete remission of other substance use disorders and a higher mortality risk among schizophrenic patients (de la Iglesia-Larrad et al., 2020). Tapering benzodiazepines in these patients was associated with improvements in some cognitive functions (de la Iglesia-Larrad et al., 2020).

Among patients with substance use disorders, the use of benzodiazepines is very high. A study showed that approximately 86% of the individuals with addictions who were admitted to a detoxification unit used benzodiazepines, 75% of them without prescription (Roncero et al., 2012). Treating insomnia in patients with drug addiction while hospitalized for detoxification is crucial, as patients with insomnia before and during admission have a higher risk of relapse after discharge (Grau-López et al., 2014).

## 3 Benzodiazepine deprescribing in clinical practice

The deprescription of benzodiazepines is a real-world challenge that should be considered in patients on prolonged and unjustified treatments, due to their association with dependence and adverse effects. Key challenges in the deprescription process include managing potential addiction to benzodiazepines, as well as addressing rebound effects and withdrawal syndrome. Withdrawal syndrome is characterized by headache, sleep disturbances, anxiety, depression, difficulty in concentration, confusion, delirium and delusions, among other manifestations (Pétursson, 1994). Long-term users or elderly people with metabolic problems are particularly susceptible to withdrawal syndrome (Jobert et al., 2021). Such long-term users are usually more reluctant to discontinue treatment, due to the anticipated withdrawal symptoms. Primary care physicians, despite their awareness of the risks associated with long-term benzodiazepine use, can find the management of withdrawal difficult. They have expressed concern about patients experiencing distress and a lack of alternatives to offer them (Sirdifield et al., 2013). This mutual hesitation can contribute to the continuation of benzodiazepine treatment. Additionally, the high workload of primary care physicians, limited consultation time, and the low availability of psychotherapy in the public healthcare system or pharmacological alternatives for addressing these barriers, further complicate deprescription management (Lasserre et al., 2010).

Strategies for successful benzodiazepine deprescription include gradual dose reductions, switching to another type of medication, or the adoption of non-pharmacological interventions, including patient education and cognitive-behavioral therapy (Brandt et al., 2024; Soni et al., 2023; Dou et al., 2019; Tannenbaum et al., 2014; Satué de Velasco, 2014). Interventions to discontinue long-term benzodiazepine use have been studied in Spanish clinical practice. Two brief structured educational interview programs delivered by general practitioners, with and without follow-up visits, were found to be more effective than standard of care in stopping long-term benzodiazepine use over 12 months (Trépel et al., 2020). Another study showed that the efficacy of interventions to reduce chronic benzodiazepine use persisted even after 3 years, with the probability of stopping benzodiazepine use being 41% higher in patients in the intervention group versus the control group (Fernandes et al., 2022).

Effective communication among the patient, pharmacist and physician is crucial for the successful prescribing and deprescribing process. The treatment plan should be individualized based on the patient characteristics and socio-environmental context. Shared decision-making with the patient encourages the latter to take responsibility and adhere to the discontinuation program.

We present two case studies that describe the course of complicated processes of benzodiazepine withdrawal observed in our clinical practice (Table 1). These cases represent profiles of patients frequently found in clinical practice. Written informed consent was obtained from the patients to share their anonymized data with research purposes. Case 1 represents a patient with reluctance to CBT-I, citing lack of time due to work commitment, but other factors may have had an influence such as lack of access to CBT-I within the Spanish healthcare system, stigma associated with mental health, a dysfunctional belief regarding benzodiazepine dependence, or a lack of motivation (Zamorano et al., 2023). Case 1 also addresses the initiation of daridorexant, a dual orexin receptor antagonist (DORA), which has a different mechanism of action to support benzodiazepine discontinuation (Álamo et al., 2024). Daridorexant is used as a non-sedative treatment of insomnia disorder with a favorable efficacy/safety profile (Mignot et al., 2022) and grade A recommendation from the European insomnia guide (Riemann et al., 2023). This patient was able to discontinue benzodiazepines after gradually reducing the dose, and improved sleep quality and hyperarousal after daridorexant initiation. Case 2 describes a patient who received CBT-I and a reduced benzodiazepine dose, with improvements in insomnia and anxiety.

**TABLE 1:** Case reports of patients with benzodiazepine treatment for insomnia.

**Case 1: Benzodiazepine withdrawal in a young woman** ***Patient information*** A 32-year-old female lawyer lives with her husband and has no children. She has had difficulty initiating and maintaining sleep for the past 8 years. She reports generalized pain that interferes with sleep, as well as anxiety and a constant state of hyperarousal. This has led to daytime sleepiness, irritability, and concentration difficulties ***Medical history*** Fibromyalgia, anxiety-depressive syndrome (under psychiatric follow-up), and hypothyroidism ***Medications*** Levothyroxine, sertraline (50 mg/day), lormetazepam (2 mg/night) ***Initial sleep assessment*** ISI: 18/28; PSQI: 14/21 (without treatment) ***Diagnosis*** Chronic insomnia and benzodiazepine dependence ***Treatment approach*** *Initial management* HDRS: 6/52. The first intervention was an attempt to reduce lormetazepam from 2 mg to 1 mg per night, together with sleep hygiene. The patient declined a structured CBT-I program due to a lack of time caused by work commitments *Follow-up: 1* *month* The dose reduction of lormetazepam to 1 mg was initially successful; however, after 3 weeks, the patient returned to the previous dose due to worsening symptoms. Daridorexant (50 mg/night) was initiated while reducing lormetazepam again to 1 mg *Follow-up: 2* *months* A reduction of lormetazepam to 1 mg per night was achieved without apparent relapse and maintained good subjective sleep quality. A complete withdrawal of lormetazepam was then proposed while maintaining daridorexant 50 mg/night *Last follow-up: 3.5* *months* The patient reported a reduction in hyperarousal, particularly at bedtime and during nocturnal awakenings. She had an improvement in ISI and PSQI scores at this visit (ISI: 9/28; PSQI: 8/21) No additional changes in her medications were necessary. The withdrawal process of lormetazepam was well-tolerated, with improvements in sleep quality and daytime functioning.with daridorexant 50 mg/night
**Case 2: Patient with insomnia with an emotional/social component** ***Patient information*** A 56-year-old man, retired from the civil guard, is divorced and has one child. He had difficulty initiating and maintaining sleep for the past 3 years, with daytime repercussions. These changes coincided with increased work responsibilities and stress while still working, and poor sleep hygiene. Since the onset of the sleep problem, he has been receiving treatment with lormetazepam, which improved both sleep and anxiety ***Medical history*** Arterial hypertension, diabetes mellitus, overweight, smoker (10 cigarettes/day) ***Medications*** Lormetazepam (1 mg 1-0-1), enalapril, metformina, and simvastatina ***Initial sleep assessment*** ISI: 15/28 Laboratory tests were normal ***Diagnosis*** Chronic insomnia and anxiety **Treatment approach** *Initial management* Gradual withdrawal of lormetazepam together with CBT-I was proposed. CBT-I included not only sleep advice but also lifestyle recommendations, such as doing physical activity and reducing stress *Follow-up: 1* *month* The lormetazepam dose at breakfast was stopped and maintained to 1 mg at dinner (0-0-1). There was a considerable improvement in anxiety and insomnia *Last follow-up: 2* *months* Lormetazepam 0.5 mg at dinner (0-0-1) was maintained (the patient’s follow-up has been coordinated with the Psychiatry Unit, which decided to continue this dose of lormetazepam)

Abbreviations: CBT-I, cognitive behavioral therapy for insomnia; HDRS, hamilton depression rating scale; ISI, insomnia severity index; PSQI, pittsburgh sleep quality index.

Scores: HDRS: higher scores indicate more severe depression (0–7: normal, 8–16: mild depression, 17–23: moderate depression, ≥24: severe depression). ISI: higher scores indicate more severe insomnia (0–7: no clinically significant insomnia, 8–14: subthreshold insomnia, 15–21: moderate insomnia, 22–28: severe insomnia). PSQI: higher scores indicate worse sleep quality (0–4: good sleep quality, >5: poor sleep quality).

## 4 Strategic proposals for the rational use of benzodiazepines in Spain

Addressing sleep education is important. Enhancing understanding of the appropriate use and the misuse of benzodiazepines can benefit both the general public and healthcare professionals now and long-term in the future. After reviewing the main articles focused on this topic we propose practical and highly relevant initiatives for the rational use of benzodiazepines in our country (Table 2). These include public education and awareness campaigns, for which social media platforms could be used, and updated training for healthcare providers.

**TABLE 2:** Strategic proposals for the rational use of benzodiazepines in Spain.

Proposals	Feasibility	References^a^
Education and awareness		
Conduct mass awareness-raising campaigns in society regarding the importance of sleep as a determining factor for good health, together with diet and physical exercise	High	Wakefield et al. (2010)
Develop and disseminate materials and tools on sleep hygiene accessible to the general population	High	Ilhan et al. (2022), Seda-Cansu and Seher (2022), Garbers et al. (2024), Irish et al. (2015)
Raise awareness among policymakers about the importance of aligning schedules with sleep physiology to encourage changes in communication and digital media	Moderate	Adornetti et al. (2025), Kelley et al. (2015), Gurubhagavatula et al. (2021), Chkhaidze et al. (2024)
Create sleep education and benzodiazepine discontinuation programs across healthcare professions, including primary care, pharmacy, nursing, and social work	High	Morbioli and Lugoboni (2021), Bourcier et al. (2018)
Implement large-scale sleep hygiene training in companies for both employees and employers	High	Redeker et al. (2019), Olson et al. (2015), Robbins et al. (2019)
Educate employers about the importance of digital detox for their employees and the impact of sleep disorders upon productivity and accidents at work	Moderate	Mizrak et al. (2025), Dresp-Langley and Hutt (2022), Ansari et al. (2024)
Direct targeted awareness programs in schools and high schools for younger populations	Moderate	Chung et al. (2017), Blunden and Rigney (2015)
Include basic aspects of sleep into school and university curricula	Moderate	Meltzer et al. (2009), Salas et al. (2018), Falloon et al. (2024), Meaklim et al. (2023)
Address the “culture of immediacy” in the population and raise awareness of its impact on health	Moderate	MacKenzie et al. (2022)
Training for healthcare professionals		
Develop specialized training programs in sleep disorders, adapted to all specialties, in short formats and oriented towards problem-solving	High	Wappel et al. (2021)
Train in the importance of conducting good diagnostic screening of insomnia to distinguish between physiological and other causes	High	Winkelman (2020)
Promote the use of therapeutic alternatives to benzodiazepines to treat sleep disorders, such as CBT-I, as well as pharmacological strategies with a more favorable efficacy/safety balance	Moderate	Riemann et al. (2023), Álamo et al. (2024)
Complement training with the development of protocols, consensus and clinical practice guidelines that can be used in daily practice	High	Manber et al. (2012)
Raise awareness among healthcare professionals about the importance of recording a complete medical history, including a brief assessment of sleep quality	High	Riemann et al. (2023), Baddam et al. (2024)
Remind healthcare professionals of the potential adverse events and risks associated with benzodiazepine use	High	Brandt and Leong (2017), Islam et al. (2016), Poly et al. (2020), Grigoriadis et al. (2020), Sun et al. (2019)
Disseminate materials aimed at health professionals on the importance of sleep for their own healthcare	High	Stewart and Arora (2019), Trockel et al. (2020), Matricciani et al. (2025)
Regulation and improvements in administration		
Disseminate existing guidelines on the use of benzodiazepines across all specialties to promote adherence	High	Tomasone et al. (2020), Silva et al. (2021)
Implement an expiration period for benzodiazepine prescriptions (e.g., 1 month, with reevaluation) and include notifications in the electronic health record/prescription system	High	Riemann et al. (2023), Ellis et al. (2023), Mekonnen et al. (2021)
Optimize collaboration between specialties through a shared consultation model, facilitating communication between specialists (psychiatry and neurology), primary care, and community pharmacy	Moderate	Furbish et al. (2017), Edinger et al. (2016)
Establish a coordinated, bidirectional communication program between physicians and community pharmacies to monitor benzodiazepine use and abuse, and to improve de-addiction processes	Moderate	Huon et al. (2023), Garcia et al. (2024)
Facilitate, from the public and private administration, access to benzodiazepine alternatives such as psychological therapy and drugs with a more favorable efficacy/safety profile, compatible with long-term use if necessary	Moderate	Álamo et al. (2024), Manber et al. (2023), Hughes (2024)
Include the collection of sleep-related information in occupational health assessments	High	Morin and Jarrin (2022)
Promote regulations that protect employee sleep health by addressing key factors such as digital detox, shift work regulation, and on-call duties	Moderate	Gurubhagavatula et al. (2021), Lerouge and Trujillo Pons (2022), Bumpstead et al. (2025)
Evidence generation		
Conduct epidemiological studies to determine the current prevalence of sleep disorders	High	NA
Develop social media listening studies to analyze conversations on social platforms regarding sleep problems and the treatments used by the population	High	NA

^a^ The proposals are supported by the references provided.

All authors graded the proposals individually. The feasibility and relevance of each proposal were graded as “moderate” or “high”. A majority was considered when three or more authors voted in favor of an option. All proposals were graded as highly relevant, and therefore, a column for “relevance” has not been included in the table.

We also include proposals for regulatory authorities that could contribute to the rational use of benzodiazepines. In Spain, there is a procedure known as “inspection validation of prescriptions,” by which the Health Services Inspection authorizes the prescription of medications and pharmaceutical products that require special control (Orozco et al., 2013). The system could help to promote the appropriate use of benzodiazepines in Spain by enforcing the use of these drugs according to the approved indications in the summary of product characteristics and the recommended use in the clinical practice guidelines (Orozco et al., 2013). Also, the regulatory authorities could facilitate access to CBT-I allowing more consultation time per patient and incorporating more physicians and psychologists to treat insomnia and sleep disorders within the Spanish healthcare system, although we recognize that implementing this will require considerable resources and time. Regulatory authorities could also improve access to new, effective and safe pharmacological treatments with a different mechanism of action, such as DORAs, that are not associated with the risks posed by benzodiazepines (Álamo et al., 2024).

Artificial intelligence (AI) and machine learning (ML) will probably transform how we diagnose and treat insomnia. Incorporating AI into sleep medicine could predict risk for sleep disorders, improve the accuracy of diagnoses and referrals, and personalize treatment (Huang and Huang, 2023; Xu et al., 2022). For instance, ML can analyze a range of sleep and clinical data to detect sleep quantity and quality, that might otherwise go unnoticed, with a view to predicting treatment response (Gabbay et al., 2024). Additionally, AI tools can help monitor the efficacy and safety of treatments, providing valuable information for managing patients. These tools represent innovation, time savings, and equitable healthcare solutions. Supervised machine learning methods, including algorithms such as decision trees and random decision forests, could be used to increase predictive accuracy within monitoring algorithms. Once these tools are validated, healthcare professionals should be trained in their use.

## 5 Conclusion

The prevalence of benzodiazepine use and misuse in Spain is high, and has been rising in the last years, with noticeable increases among vulnerable populations of both adolescents and older adults. The long-term use of these drugs is associated with adverse effects and dependence, and therefore, their use should be restricted to their approved indications and according to the recommendations of the clinical guides (i.e., less than 4 weeks). We advise avoiding the indiscriminate and long-term use of benzodiazepines, and recommend the adoption of pharmacological or non-pharmacological therapeutic alternatives instead. With this in mind, we propose several strategies that could be implemented in Spain, with variable levels of feasibility. These strategies include education and awareness-raising campaigns for the general population, specialized training for healthcare professionals, structural and organizational changes promoted by public and private administrations, and access to new therapeutic options with a better efficacy/safety profile. We anticipate that the implementation of these proposals would reduce the current unmet needs in the clinical management of insomnia, and improve the management and quality of life of patients.

## Data Availability

The original contributions presented in the study are included in the article/supplementary material, further inquiries can be directed to the corresponding author.
